# AVA Spring Meeting and AGM, University of Bradford, 26 March 2018

**DOI:** 10.1177/2041669518791669

**Published:** 2018-08-24

## Binocular Summation: A Meta-Analysis of 65 Studies

Daniel H. Baker and Freya A. Lygo

Department of Psychology, University of York, York, UK

Tim S. Meese and Mark A. Georgeson

School of Life and Health Sciences, Aston University, Birmingham, UK

### 

Binocular summation is the advantage in contrast sensitivity when using two eyes versus one. It has been widely studied owing to its clinical importance as a measure of binocular function, and because the precise level of summation is determined by the magnitude of nonlinearities in the early visual system, before binocular combination. However, most studies have involved small sample sizes, making exact estimation problematic. We conducted a meta-analysis of 65 studies reporting psychophysical estimates of binocular summation in 716 observers. The lower bound of the 95% confidence interval on the mean summation ratio was consistently above the canonical value of $\sqrt{2}$, regardless of how studies were weighted. We further explored how methodological factors affect summation estimates, both by using subsets of the meta-analysis data and also confirming with stand-alone studies. These analyses show that stimulus factors such as spatiotemporal frequency affect summation, and that the imbalance in sensitivity across the eyes can moderate summation estimates. We suggest that there is no single canonical value for binocular summation, but that instead it takes on a range of values between approximately $\sqrt{2}$ and 2, depending on stimulus properties. In addition, when the two eyes are not balanced, summation estimates are reduced when calculated relative to the threshold of the more sensitive eye, but can be slightly elevated when the mean monocular threshold is used. Future studies can obtain accurate summation estimates by normalising monocular contrasts to account for sensitivity differences or by modelling results using a simple two-parameter model of binocular combination.

## Ipsilateral Sensitivity to Visual Motion Is Restricted to V5/MT+ in the Right Cerebral Hemisphere

Samantha L. Strong

School of Optometry and Vision Science, University of Bradford, UK

Edward H. Silson

National Institute of Mental Health, Bethesda, USA

André D. Gouws and Antony B. Morland

York Neuroimaging Centre, York, UK

Declan J. McKeefry

School of Optometry and Vision Science, University of Bradford, UK

### 

Previous experiments have demonstrated that transcranial magnetic stimulation (TMS) of human V5/MT+ in the right cerebral hemisphere can induce deficits in visual motion perception in both the contra- and ipsi-lateral visual hemi-fields. However, when TMS is applied to left V5/MT+, motion deficits are restricted to the contra-lateral hemi-field (Thakral and Slotnick, 2011).

One possible explanation for this may lie in differential stimulation of sub-divisions within V5/MT+ across the two hemispheres. V5/MT+ has two major sub-divisions: MT/TO-1 and MST/TO-2, and the latter sub-division contains neurons with large receptive fields (RFs) that extend much further into the ipsi-lateral hemi-field (up to 15°) than MT/TO-1.

We wanted to re-examine this functional asymmetry between V5/MT+ across both hemispheres by using TMS. MT/TO-1 and MST/TO-2 were identified in six subjects using specialised fMRI localisers, and centre-of-mass co-ordinates were used as target points for the TMS experiment (70% strength; 25 Hz; 200 ms). Subjects identified the translational direction (up/down) of coherently moving dots presented in either the left or the right visual field whilst TMS pulses were applied synchronously with stimulus onset.

Application of TMS to MT/TO-1 and MST/TO-2 in the right hemisphere affected ability to perceive direction of translational dots in both the contra-lateral and ipsi-lateral visual fields, whereas detrimental effects following application of TMS to MT/TO-1 and MST/TO-2 in the left hemisphere were restricted to the contra-lateral visual field.

This result suggests an enhanced role for the right hemisphere in processing full-field translational motion, but contrary to our hypothesis, effects differ across hemispheres rather than within sub-divisions of V5/MT+.

### 

ReferenceThakral, P. P., &
Slotnick, S. D. (2011). Disruption of MT impairs motion processing.
*Neuroscience letters*, *490*(3),
226–230.10.1016/j.neulet.2010.12.05721195742

## Human S-Cone Electroretinograms Obtained by Silent Substitution Stimulation

J. Maguire

School of Optometry and Vision Science, University of Bradford, UK

N. R. A. Parry

School of Optometry and Vision Science, University of Bradford, UK; Vision Science Centre, Manchester Royal Eye Hospital, Central Manchester University Hospitals NHS Foundation Trust, Manchester Academic Health Science Centre, Manchester, UK; Faculty of Biology, Medicine & Health, University of Manchester, UK

J. Kremers

School of Optometry and Vision Science, University of Bradford, UK; Department of Ophthalmology, University Hospital Erlangen, Germany

I. J. Murray

Faculty of Biology, Medicine & Health, University of Manchester, UK

D. McKeefry

School of Optometry and Vision Science, University of Bradford, UK

### 

The purpose of this study was to characterise the response properties of the human S-cone electroretinogram (ERG) in normal trichromats in both time and frequency domains. We also examined how the S-cone response was affected in Blue Cone Monochromatism (BCM) and Enhanced S-cone Syndrome (ESCS). We recorded S-cone-mediated ERGs using steady-state (sinusoidal) and transient (square wave) silent substitution stimuli. Responses were obtained from n = 16 normal trichromatic observers as well as two subjects with BCM and one with ESCS.

Temporal response functions were obtained using steady-state sinusoidal, S-cone isolating stimuli (1000 Td, cone contrast = 0.25) which varied in temporal frequency between 5 Hz and 75 Hz. The functions obtained were approximately low pass in nature and response amplitudes fell below threshold criterion above 30 Hz. The S-cone ERGs elicited by transient stimuli were characterised by components with implicit times longer than those measured for the corresponding components in the L- and M-cone ERGs. In normal trichromats, the S-cone response lacked a prominent positive offset d-wave which was observed in the L- and M-cone responses. The S-cone ERGs obtained from the BCM and ESCS patients did exhibit a more prominent offset response.

The results demonstrate that silent substitution stimuli can be used to generate ERGs that selectively reflect S-cone-mediated vision in humans. S-cone ERGs have response properties that are different to those mediated by L- and M-cones. Furthermore, the results raise the possibility that differences in the ERG waveforms observed in retinal pathologies that affect the S-cone system may reflect a re-organisation of S-cone signal processing in these conditions.

## Cognitive Demand and Accommodative Microﬂuctuations

Niall J. Hynes and Matthew P. Cufflin

School of Optometry and Vision Science, University of Bradford, UK

Karen M. Hampson

Department of Engineering Science, University of Oxford, UK

Edward A. H. Mallen

School of Optometry and Vision Science, University of Bradford, UK

### 

When viewing a static target, the accommodation response fluctuates. These variations in response are termed microfluctuations. Microfluctuations can be divided into two categories: the Low Frequency Component (LFC) measuring below 0.6 Hz and the High Frequency Component (HFC) measuring between 1.0 and 2.3 Hz. This experiment aims to investigate the effect that mental cognition has on the nature of accommodative microfluctuations. The study consisted of 22 participants (mean age: 25.96 ± 4.99, range: 20–35 years) comprising 11 emmetropes and 11 myopes. Accommodation was monitored continuously using a modified Shin Nippon SRW-5000 autorefractor whilst participants completed three tasks of varying cognitive demand. Participants completed these tasks in a randomised order:

(i) Reading numbers aloud (Num).

(ii) Simple arithmetic (SA).

(iii) Complex arithmetic (CA).

Data were analysed using Fast Fourier Transform functions in Matlab. A repeated measures analysis of variance highlighted a significant main effect in the mean power of the HFC, *F*(2, 42) = 10.03, *p* < .01. Pairwise analyses revealed that these differences exist between SA and CA (*p* < .01), and the Num and CA (*p* < .01) conditions with the HFC power being highest for the CA condition. No significant differences were found in power of the LFC across conditions. Whilst cognitive demand has no effect on the overall accommodative response or the LFC in accommodative microfluctuations, it does appear to increase the power of the HFC during complex arithmetic. As the HFC has been shown to be associated with arterial pulse, this may correspond with other physiological finding related to cognitive demand.

## The McGill Face Database: A Novel Database of Facial Expressions of Mental States

Gunnar Schmidtmann

Eye & Vision Research Group, School of Health Professions, University of Plymouth, Plymouth, UK

Ian Gold

Departments of Philosophy and Psychiatry, McGill University, Montreal, Quebec, Canada

### 

Current databases of facial expressions of mental states typically represent only a small subset of expressions, usually covering the basic emotions (fear, disgust, surprise, happiness, sadness and anger). To overcome these limitations, we introduce a large new database of pictures of facial expressions reflecting the richness of mental states. Ninety-three expressions of mental states were interpreted by two professional actors and high-quality pictures were taken under controlled conditions in front and side view. The database was validated with two different experiments (N = 65). First, a four-alternative forced choice paradigm was employed to test the ability of participants to correctly select a term associated with each picture. Second, the observers’ task was to indicate the point within an emotional space ranging from pleasant to unpleasant in one dimension and arousal high to arousal low in the other. Results from both experiments demonstrate that subjects can reliably recognise such a huge diversity of emotional states from facial expressions. The McGill Face Database provides a wide range of facial expressions that can be linked to mental state terms and can be accurately characterised in terms of arousal and valence independent of terms. The database is also available in French and German and is freely available for scientific, non-commercial purposes.

## The Effects of Long-Term Visual Experience on Peripheral Motion Sensitivity

Eilidh Fenner and Alexandra Levine

University of York

Charlotte Codina and David Buckley

University of Sheffield

Heidi Baseler

University of York

### 

Catching a ball, deflecting an opponent and being aware of potential threats in one’s surroundings all depend on peripheral visual sensitivity. Sports, video gaming and deafness have the potential to shape long-term visual experience by placing particular demands on peripheral vision. The purpose of the current study was to compare the effects of visual experience across sports, video gamer, early deaf and control groups using the same motion detection task. Participants indicated which of two clusters of dots presented on either side of fixation contained motion. Threshold (minimum speed required to detect motion) and reaction time (RT) were recorded for dot cluster pairs centred at various eccentricities between 5° and 40° along the horizontal meridian. Both threshold and RT increased with eccentricity across groups. Thresholds varied significantly between groups at 20° and 40°, but not at more central eccentricities. Threshold and RT were affected differently across groups, for example, peripheral thresholds were lowest in the deaf group, while RTs were lowest in the sports group. Higher self-reported stress levels also correlated with lower thresholds in all groups. Our results suggest that differences in long-term visual experience, through deafness or practicing certain hobbies, modify visual motion sensitivity in different ways.

### Funding

Funding provided by the Laidlaw Scholarship.

## The Threshold for Visually Induced Illusions (the McGurk Effect) Decreases With Development

Rebecca J. Hirst

University of Nottingham, Nottingham, UK

Jemaine E. Stacey

Nottingham Trent University, Nottingham, UK

Lucy Cragg

University of Nottingham, Nottingham, UK

Paula C. Stacey

Nottingham Trent University, Nottingham, UK

Harriet A. Allen

University of Nottingham, Nottingham, UK

### 

The McGurk effect is a visually induced illusion in which a seen mouth movement changes the sound perceived. The influence of vision over audition is proposed to increase across development such that vision plays an increasingly important role in perception (Nava & Pavani, 2013). In this study, we assessed the impact of auditory and visual noise on the McGurk effect in 32 adults (aged 20–35 years) and 90 children (aged 3–12 years). We predicted that susceptibility to the McGurk effect would increase with age in children, and also that adults will be more susceptible to the McGurk effect. Furthermore, we predicted the threshold for the McGurk effect (i.e., the level of auditory noise required to induce the effect and visual noise required to abolish it) would be lower in adults compared with children. In line with our predictions, we found that susceptibility to the McGurk effect increased with development and was higher in adults than children. Auditory noise increased the likelihood of vision changing auditory perception, and visual noise reduced the likelihood of vision changing auditory perception. Children also required more auditory noise than adults to induce McGurk responses and less visual noise compared with adults to reduce McGurk responses (i.e., adults and older children were more easily influenced by vision). Reduced susceptibility to visually induced illusions in childhood supports the theory that sensory dominance shifts across development.

### 

ReferenceNava, E., & Pavani, F.
(2013). Changes in sensory dominance during childhood: Converging
evidence from the Colavita effect and the sound-induced flash
illusion. *Child Development*,
*84*(2), 604–616. doi:
10.1111/j.1467-8624.2012.01856.x.10.1111/j.1467-8624.2012.01856.x23006060

## Quantifying the Effect of Viewpoint Changes on Sensitivity to Face Identity

Alexander G. Swystun, James Heron and Andrew J. Logan

School of Optometry and Vision Science, University of Bradford, UK

### 

Faces can be recognised across different viewpoints. Previous reports, however, suggest that changes in viewpoint reduce face identification accuracy. We aimed to quantify the effect of variations in viewpoint on the ability to discriminate between face identities. Discrimination thresholds were measured for three observers for synthetic faces shown from the same frontal view (baseline). These baseline thresholds were compared to those for the same synthetic faces presented with a change in viewpoint (5°, 10° or 20°). Three different types of viewpoint change were tested: (a) front side (frontal face matched to 20° side view), (b) symmetrical (10° right to 10° left) and (c) asymmetrical (5° left to 15° right). Relative to baseline, viewpoint changes significantly increased discrimination thresholds. The magnitude of this reduction in sensitivity increased monotonically with the size of viewpoint change (front-side: 5° = 1.22 ×, 10° = 1.87 ×, 20° = 2.21×). Importantly, the effect of the three types of viewpoint change was not equivalent: While a symmetrical 10° change did not significantly reduce sensitivity (1.11×), asymmetrical changes in viewpoint, of the same magnitude, increased face discrimination thresholds by a factor of 1.93×. Changes in viewpoint significantly reduce discrimination sensitivity for face identity. The magnitude of this reduction in sensitivity is related to both the size and type of viewpoint change. These results suggest that the neural mechanisms that encode face identity are also tuned to viewpoint.
